# Photocycloaddition of aromatic and aliphatic aldehydes to isoxazoles: Cycloaddition reactivity and stability studies

**DOI:** 10.3762/bjoc.7.18

**Published:** 2011-01-26

**Authors:** Axel G Griesbeck, Marco Franke, Jörg Neudörfl, Hidehiro Kotaka

**Affiliations:** 1 University of Cologne, Department of Chemistry, Organic Chemistry, Greinstr. 4, D-50939 Köln, Germany; Fax: +49(221)470 5057

**Keywords:** isoxazoles, oxetanes, Paternò–Büchi reaction, photochemistry

## Abstract

The first photocycloadditions of aromatic and aliphatic aldehydes to methylated isoxazoles are reported. The reactions lead solely to the *exo*-adducts with high regio- and diastereoselectivities. Ring methylation of the isoxazole substrates is crucial for high conversions and product stability. The 6-arylated bicyclic oxetanes **9a**–**9c** were characterized by X-ray structure analyses and showed the highest thermal stabilities. All oxetanes formed from isoxazoles were highly acid-sensitive and also thermally unstable. Cleavage to the original substrates is dominant and the isoxazole derived oxetanes show type T photochromism.

## Introduction

Photochemical [2 + 2] cycloadditions are among the most efficient photoreactions and are used in numerous synthetic applications due to the generation of highly reactive four-membered rings. An important example is the photocycloaddition of electronically excited carbonyl compounds to alkenes (Paternò–Büchi reaction). This reaction is a superior route to oxetanes, which can be subsequently transformed into polyfunctionalized products [1]. With regards to the regio- and diastereoselectivity of the Paternò–Büchi reaction, recent experimental and computational studies have brought about a remarkable increase in our understanding of this reaction. Especially the role of intermediary triplet 1,4-biradicals – their stability, lifetimes and intersystem crossing geometries – was crucial for a more sophisticated description [2–5], which also improved the synthetic significance of this reaction [6].

Previous publications have clearly demonstrated the versatility of the Paternò–Büchi reaction in various synthetic applications which gives rise to a multiplicity of different products. The photocycloaddition of furans to carbonyl compounds affords the corresponding β-hydroxy-1,4-diketones after hydrolysis of the primary photochemical products (photo aldol reaction) [7], whilst the reaction of oxazoles with carbonyl compounds is a convenient protocol for the stereoselective synthesis of α-amino β-hydroxy ketones [8–9] as well as highly substituted α-amino β-hydroxy acids [10–11].

The results on five-membered aromatic heterocycles published so far, however, has not included a study of isoxazoles as substrates in the Paternò–Büchi reaction. This class of heterocyclic compounds can be considered as masked β-amino ketones [12], and subsequently hydrolysed to the corresponding 1,3-diketones [13] or deaminated to yield Michael systems [14]. Thus, isoxazoles also appear to be important substrates for carbonyl–ene photocycloaddition due to possible applications in ring-opening transformations.

## Results and Discussion

### Synthesis of the isoxazole substrates

**Scheme 1:**
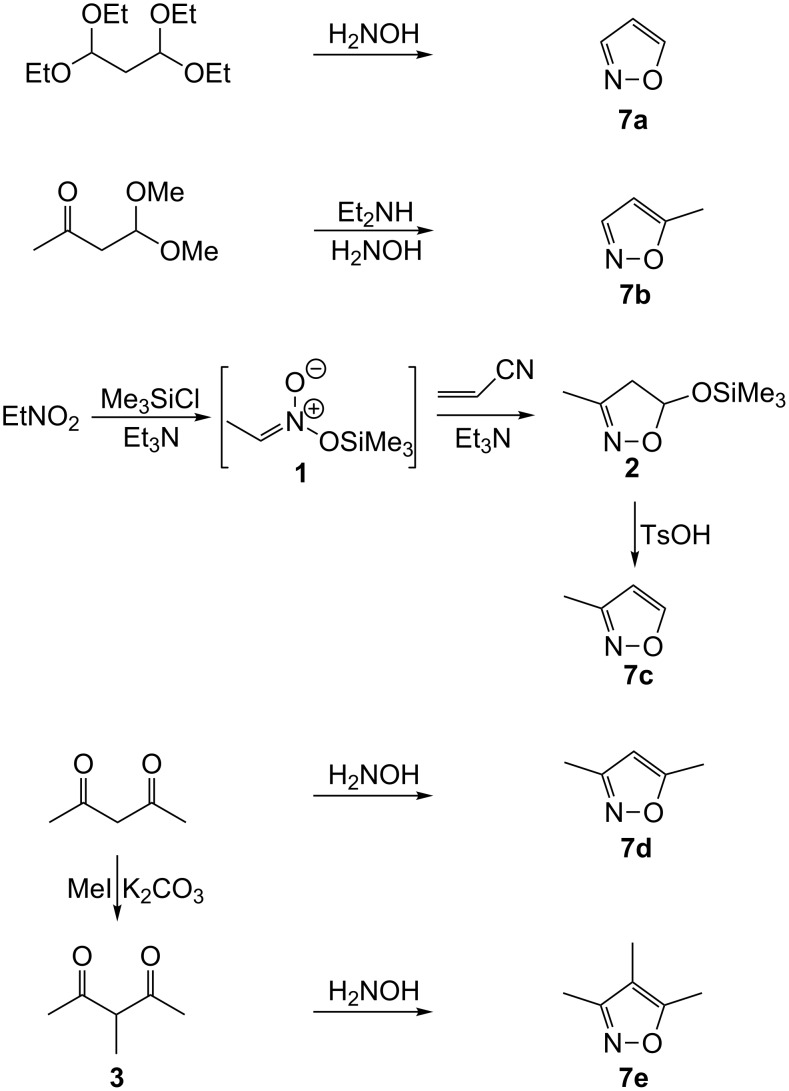
Synthetic routes to isoxazoles **7a**–**7e**.

The substrates isoxazole (**7a**), 5-methylisoxazole (**7b**), 3,5-dimethylisoxazole (**7d**) and 3,4,5-trimethylisoxazole (**7e**) were synthesized by reaction of the corresponding carbonyl compounds with hydroxylamine, while 3-methylisoxazole (**7c**) was obtained by the [3 + 2]-cycloaddition of acrylonitrile with the trimethylsilylester of *aci*-nitroethane **1** (Scheme 1). The reaction of acetylacetaldehyde with hydroxylamine gave **7b**, exclusively.

**Scheme 2:**
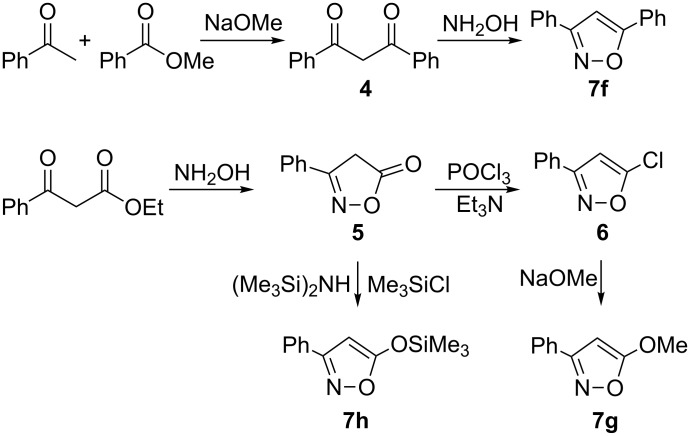
Synthetic routes to isoxazoles **7f**–**7h**.

3,5-Diphenylisoxazole (**7f**) was prepared from acetophenone and methyl benzoate, followed by cyclization of the resulting diketone **4** with hydroxylamine. 5-Methoxy-3-phenylisoxazole (**7g**) and 5-(trimethylsilyloxy)-3-phenylisoxazole (**7h**) were synthesized from 3-phenylisoxazol-5-one (**5**) which was obtained by the reaction of ethyl benzoylacetate and hydroxylamine (Scheme 2). The preparation of aliphatic substituted isoxazole ethers however, could not be achieved, since the corresponding isoxazolones were unstable.

### Photochemistry of the isoxazoles **7a**–**h**: test reactions

**Scheme 3:**
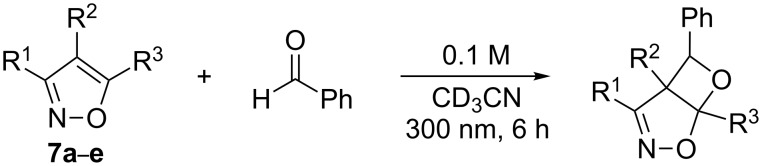
Benzaldehyde photocycloaddition to **7a**–**7e**.

The isoxazoles **7a**–**e** were irradiated in the presence of benzaldehyde or propionaldehyde as model compounds for aromatic and aliphatic carbonyl compounds, respectively, at λ = 300 nm in perdeuterated acetonitrile. ^1^H NMR studies showed that the expected photoadducts were formed only from isoxazoles **7d** and **7e** with benzaldehyde (Scheme 3 and Table 1). In the presence of propionaldehyde no reaction was observed.

**Table 1:** Irradiation of isoxazoles **7a**–**e** with benzaldehyde.

	R^1^	R^2^	R^3^	conversion [%]^a^

**7a**	H	H	H	0
**7b**	H	H	Me	0
**7c**	Me	H	H	0
**7d**	Me	H	Me	13
**7e**	Me	Me	Me	41

^a^based on the formation of the photoproduct, by NMR (benzaldehyde - isoxazole ratio = 1:1, irradiation time: 6 h).

The use of a tenfold excess of aldehyde had no significant influence on the reaction. The use of a tenfold excess of the isoxazole, however, led to a considerable change in the reaction conversions (Table 2).

**Table 2:** Irradiations of **7a**–**e** with a tenfold excess of isoxazoles.

	R^1^	R^2^	R^3^	conversion [%]^a^

**7a**	H	H	H	<5
**7b**	H	H	Me	15
**7c**	Me	H	H	10
**7d**	Me	H	Me	40
**7e**	Me	Me	Me	98

^a^based on the formation of the photoproduct, by NMR (benzaldehyde - isoxazole ratio = 1:10, irradiation time: 6 h).

The conversion is highly dependent on the degree of substitution of the isoxazole used. In terms of frontier orbital interactions, the reason is the decreasing energy difference between the HOMO of the isoxazole and the SOMO of the excited aldehyde with increasing degree of substitution. Indirect proof of the increasing energy levels of the isoxazole-HOMO is provided from the corresponding ionization energies (Table 3) [15] which decrease with increasing substitution.

**Table 3:** Vertical ionization energies (E_iv_) of isoxazoles **7a**, **7b** and **7d**.

	E_iv_ [eV]

**7a**	10.15
**7b**	9.61
**7d**	9.34

In contrast, the use of a tenfold excess of isoxazole in presence of propionaldehyde did not lead to an increased formation of the corresponding photoadducts. Only in the case of **7e** could traces of the expected photoproduct be detected (<5%). Since the LUMO energy of propionaldehyde is larger than that of benzaldehyde, it can be assumed that the energy difference between the isoxazole-HOMO and the aldehyde-LUMO is too large to promote an efficient reaction.

**Scheme 4:**
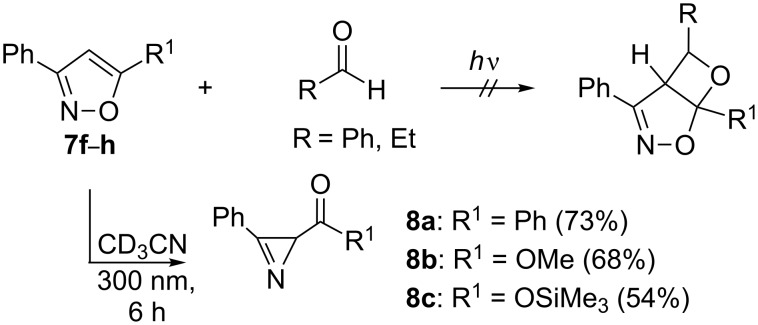
Photochemical ring contraction of isoxazoles **7f**–**7h**.

The isoxazoles **7f**–**h** were treated similarly to **7a**–**e**. However, in these experiments, the formation of the corresponding Paternò–Büchi products were not observed, neither in the presence of propionaldehyde nor in the presence of benzaldehyde. Instead, a reaction could be observed which also occurred both in the presence of a tenfold excess of aldehyde or without any aldehyde. This reaction was identified as the intramolecular ring contraction of **7f**–**h** to yield the corresponding azirines **8a**–**c** (Scheme 4) [16].

Surprisingly, in the absence of aldehydes as the potential reaction partners, the conversions of **7f**–**h** were significantly lower, suggesting the possibility of an energy transfer from the excited singlet or triplet aldehyde to the isoxazole.

**Scheme 5:**
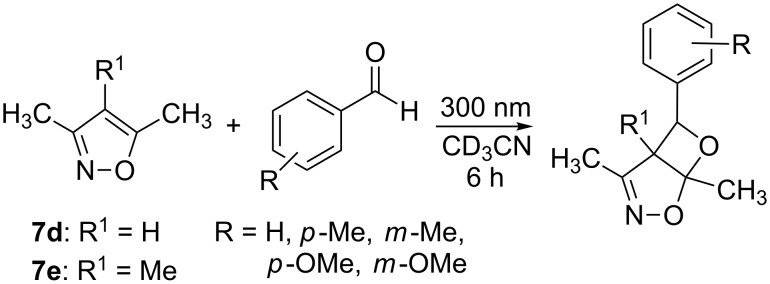
Photocycloaddition of aromatic aldehydes to di- and trimethyl isoxazoles **7d** and **7e**.

Since the photolysis of **7d** and **7e** in presence of benzaldehyde showed the highest conversions, further irradiations were conducted in order to examine the effect of other aryl substituted aldehydes on reaction conversions (Scheme 5, Table 4).

**Table 4:** Photocycloadditions of **7d** and **7e** with aromatic aldehydes.

	R	conversion [%]^a^

**7d**	H	40
“	*p*-Me	18
“	*m*-Me	18
“	*p*-OMe	<5
“	*m*-OMe	0
**7e**	H	98
“	*p*-Me	96
“	*m*-Me	92
“	*p*-OMe	65
“	*m*-OMe	19

^a^based on the formation of the photoproduct (aldehyde - isoxazole = 1:10, irradiation time: 6 h).

The reactions of **7e** with *p*- and *m*-tolualdehyde showed no change in the reaction conversions compared with benzaldehyde, whilst the conversions of **7d** with these two aldehydes were considerably decreased. Irradiation of **7d** and **7e** with *p*- and *m*-anisaldehyde showed in all cases lower reaction conversions.

### Synthesis of oxetanes from **7e** and aryl substituted aldehydes

**Scheme 6:**
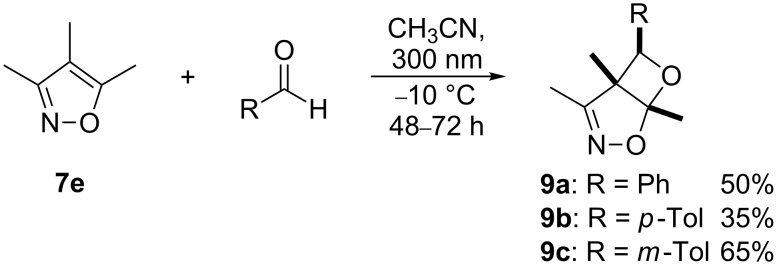
Preparative photocycloadditions of **7e** with aromatic aldehydes.

The preparative photoreactions of **7e** together with aryl substituted aldehydes were carried out in acetonitrile at −10 °C in presence of 10 mol % potassium carbonate (in order to neutralize traces of acid). In all cases, the regioisomers **9a**–**c** were formed with excellent (*exo*) diastereoselectivity (> 99:1, by ^1^H NMR spectroscopy) in moderate yields and high purities (Scheme 6).

**Figure 1:**
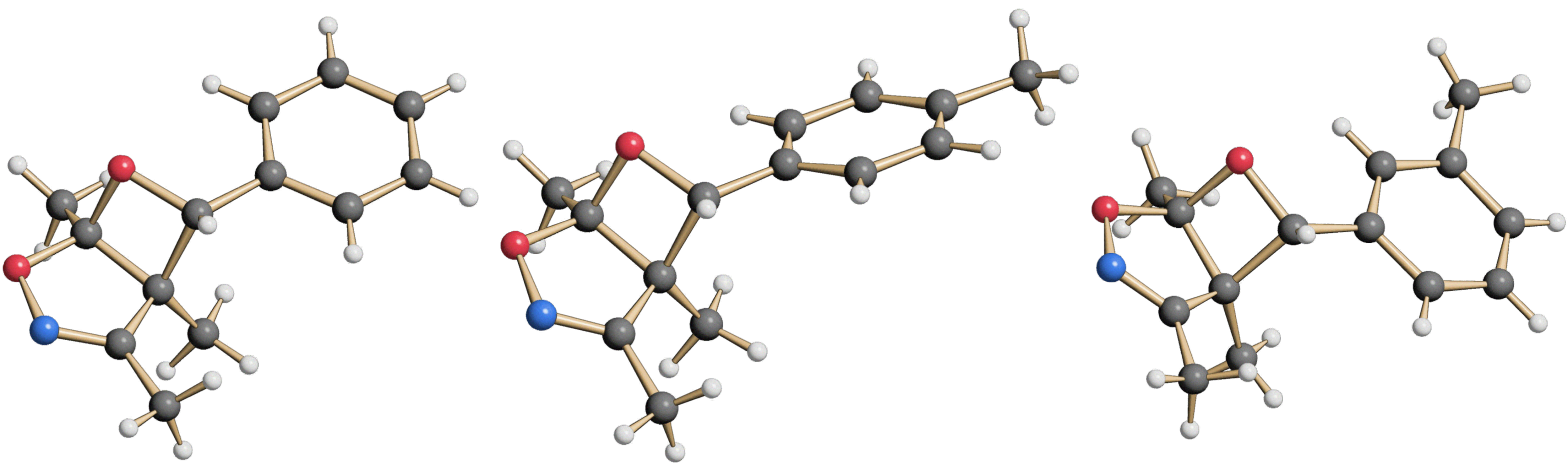
Structures of the photoproducts **9a**–**9c** in the crystal.

The chemical structures of these bicyclic oxetanes were established on the basis of the NMR- and X-ray data (Figure 1) [17].

Both regio- and diastereoselectivity are in accord with the rules previously reported for the carbonyl-furan photocycloaddition [3]: High regiocontrol due to the FMO-controlled formation of the corresponding triplet 1,4-biradical and high stereocontrol due to SOC-controlled crossing from the triplet to the singlet surface [4–5].

### Reaction behavior of the photoproducts **9a**–**c**

**Scheme 7:**
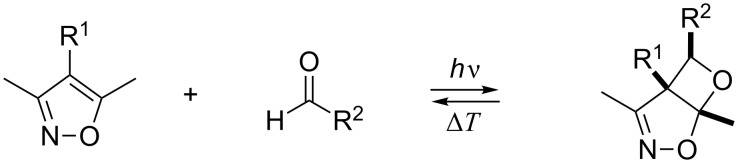
T-type photochromism of isoxazole–aldehyde pairs.

All bicyclic oxetanes obtained in the analytical photochemical experiments as well as in preparative studies (i.e., **9a**–**c**) were acid labile and decomposed already in the presence of catalytic amounts of acid to give solely the starting materials. The photoproducts are also thermally labile and decompose at temperatures above approximately 100 °C, again to give solely the starting materials. Thus, isoxazole-carbonyl photocycloaddition products constitute another class of photochromic T-type systems (Scheme 7) [18].

**Scheme 8:**
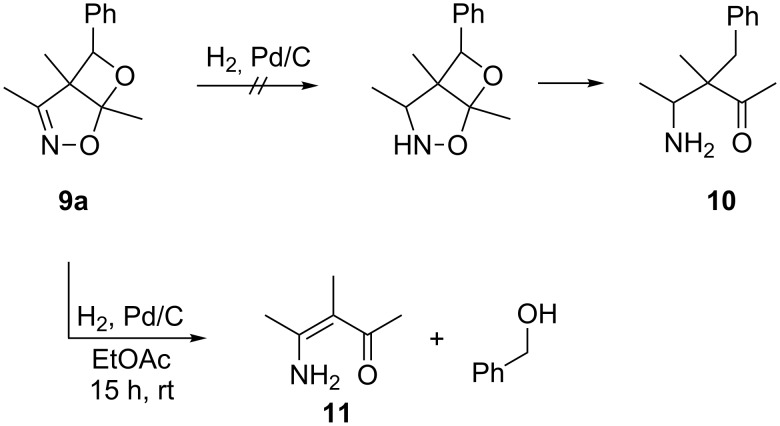
Reductive cleavage of the trimethylisoxazole adduct **9a**.

Hydrogenation of **9a** by palladium/charcoal did not lead to the expected aminoketone **10**, but to the enamino ketone **11** and benzyl alcohol, indicating decomposition of **9a** back to the starting materials, followed by hydrogenation of these substrates (Scheme 8). Variations of reaction temperature (−10 °C), reaction time (6 h, 1 h) and solvent (ethanol, ethyl acetate) led to the same results.

Hydrogenation of **9a** by Raney-nickel led to partial decomposition without any further reaction, whilst treatment with lithium aluminium hydride (3 equiv) yielded complex mixtures with benzyl alcohol as one of the main components. By contrast, no reaction could be observed in presence of sodium borohydride or sodium cyanoborohydride. Attempted reduction with sodium triacetoxyborohydride led to decomposition, probably due to traces of acetic acid contained in the hydride reagent. Reductive treatment with sodium or samarium diiodide also led to decomposition of the photoproduct. Treatment of **9a** with ethylmagnesium bromide did not lead to the alkylated photoproduct, but to partial decomposition into isoxazole **7e** and benzaldehyde, while at −78 °C, no reaction could be observed. In contrast, the use of an excess of Grignard reagent (3 equiv) at −78 °C again led to decomposition. The application of boron trifluoride at −78 °C also led to decomposition, followed by a normal nucleophilic attack of the Grignard reagent on the liberated benzaldehyde.

## Conclusion

The photocycloaddition of electronically excited carbonyl compounds to isoxazoles is clearly less effective than with other five-membered aromatic or non-aromatic heterocycles (furans, thiophenes, pyrroles, oxazoles, dihydrofurans, dihydropyrroles) [1]. Only the combination of methylated isoxazoles and aromatic aldehydes is sufficient to allow photochemical addition and give adducts with sufficient thermal stability. It cannot be excluded at the current stage, that other combinations are also reactive in Paternò–Büchi chemistry resulting in thermally labile cycloadducts which thus have not yet been detected. Apparently, the bicyclic oxetanes isolated in this study are acid-labile and thermally unstable and thus constitute a new class of T-type chromophoric systems.

## Experimental

**General methods.** All solvents were dried before use. Benzene, toluene and chloroform were distilled from CaH_2_. ^1^H NMR and ^13^C NMR spectra were recorded on a Bruker AV 300 or a Bruker DRX 500 spectrometer. Melting points were determined with a Büchi melting apparatus (type Nr. 535) and are uncorrected. X-ray data collections were performed on a Nonius Kappa-CCD-diffractometer, using a monochromatic Mo K_α_ (0,71073 Å) radiation. Combustion analyses were measured using an Elemental Vario EL Instrument. All irradiations were carried out in quartz vessels. Photochemical reactors LZ-C4 (14 × 3000 Å lamps, λ = 300 ± 10 nm) and RPR-208 (8 × 3000 Å lamps, λ = 300 ± 10 nm) were used for irradiations.

**Trimethylsilyl ester of *aci*-nitroethane (1)** [19]**.** Chlorotrimethylsilane (43.20 g, 0.4 mol, 50.5 mL) was added to nitroethane (30.0 g, 0.4 mol, 28.7 mL) and triethylamine (40.40 g, 0.4 mol, 55.6 mL) in benzene (200 mL). The mixture was stirred vigorously for 18 h at rt, filtered and evaporated. The crude product was obtained as a yellow oil and used immediately without further purification.

**3-Methyl-5-trimethylsilyloxy-2-isoxazoline (2)** [19]**.** A mixture of acrylonitrile (10.61 g, 0.2 mol, 13.2 mL), triethylamine (10.12 g, 0.1 mol, 13.9 mL) and **1** (28.73 g, 0.2 mol) in toluene (90 mL) was heated at 85 °C for 1 h. The solvent was removed and the crude product fractionated (105–110 °C, 20 mbar) to give 22.50 g (65%) of **2** as a colorless oil. ^13^C NMR (75.5 MHz, CDCl_3_): 155.2 (1C, C_q_), 97.4 (1C, CH), 47.4 (1C, CH_2_), 12.9 (1C, CH_3_), 0.0 (3C, 3 × CH_3_). ^1^H NMR (300 MHz, CDCl_3_): 5.80–5.77 (dd, 1H, ^3^*J* = 5.8 Hz, 0.9 Hz, CH), 3.04–2.96 (dq, 1H, ^3^*J* = 5.5 Hz, 0.8 Hz, CH_2_), 2.71–2.65 (dq, 1H, ^3^*J* = 1.8 Hz, 0.8 Hz, CH_2_), 2.01 (s, 3H, CH_3_), 0.15 (s, 9H, 3 × CH_3_).

**3-Methylpentane-2,4-dione (3)** [20]**.** A mixture of freshly distilled 2,4-pentanedione (28.03 g, 0.28 mol, 28.8 mL), anhydrous potassium carbonate (38.70 g, 0.28 mol) and methyl iodide (49.68 g, 0.35 mol, 21.8 mL) in acetone (200 mL) was refluxed in the dark for 20 h and then cooled to rt. The insoluble material was removed by filtration and washed thoroughly with acetone. The combined filtrate and acetone washings were then concentrated, extracted two times with chloroform, dried over magnesium sulfate and filtered. The solvent was evaporated and the residual oil distilled (170 °C) to give 23.01 g (72%) of **3** as a colorless oil. ^13^C NMR (75.5 MHz, CDCl_3_): 204.8 (2C, C_q_), 190.1 (1C, C_q_), 104.5 (1C, C_q_), 61.4 (1C, CH), 28.4 (2C, CH_3_), 23.0 (1C, CH_3_), 20.9 (1C, CH_3_), 12.2 (1C, CH_3_). ^1^H NMR (300 MHz, CDCl_3_): 3.59 (q, 1H, ^3^*J* = 7.2 Hz, CH), 2.06 (s, 6H, 2 × CH_3_), 1.97 (s, 3H, CH_3_), 1.70 (s, 1.5H, CH_3_), 1.19–1.16 (d, 3H, ^3^*J* = 6.9 Hz, CH_3_).

**1,3-Diphenylpropane-1,3-dione (4).** A mixture of acetophenone (30.04 g, 0.25 mol, 29.2 mL), methyl benzoate (34.04 g, 0.25 mol, 31.5 mL), sodium methoxide (16.21 g, 0.3 mol) and toluene (300 mL) was refluxed for 3 h. The resulting solution was concentrated, cooled to rt, poured into hydrochloric acid (300 mL, 6 N) and stirred for 30 min. The aqueous mixture was extracted two times with toluene and the combined organic layers were neutralized with aqueous sodium hydroxide. The organic solution was then washed two times with water, dried over magnesium sulfate, filtered and evaporated. The resulting solid was recrystallized twice from ethanol to yield 6.34 g (41%) of **4** as colorless crystals. ^13^C NMR (75.5 MHz, CDCl_3_): 185.6 (2C, C_q_), 135.4 (2C, C_q_), 132.4 (2C, CH), 128.6 (4C, CH), 127.1 (4C, CH), 93.0 (1C, CH). ^1^H NMR (300 MHz, CDCl_3_): 8.03–7.99 (m, 4H, CH), 7.59–7.47 (m, 6H, CH), 6.87 (s, 1H, CH).

**3-Phenylisoxazol-5-one (5)** [21]**.** A mixture of ethyl benzoylacetate (15.38 g, 80 mmol), hydroxylamine hydrochloride (5.56 g, 80 mmol), potassium carbonate (5.53 g, 40 mmol), ethanol (40 mL) and water (40 mL) was stirred at rt for 15 h. The solid was filtered, washed with water and extracted three times with ether. The combined organic layers were dried over magnesium sulfate, filtered and the solvent was evaporated. The residue was recrystallized from ethanol to give 9.21 g (71%) of **5** as colorless crystals. ^1^H NMR (300 MHz, CDCl_3_): 7.69–7.67 (m, 2H, CH), 7.54–7.46 (m, 3H, CH), 3.80 (s, 2H, CH_2_).

**3-Phenyl-5-chloroisoxazole (6)** [22]**.** A mixture of **5** (4.84 g, 30 mmol) and phosphorous oxychloride (16.3 mL, 175 mmol) was stirred at 0 °C and triethylamine (3.37 g, 33 mmol, 4.6 mL) added slowly (*T* < 25 °C). The solution was then heated at 120 °C for 2.5 h and the excess phosphorous oxychloride removed in vacuo. The brown residue was triturated with 100 mL iced water and extracted two times with ethyl acetate. The combined organic layers were dried over magnesium sulfate, filtered and the solvent was evaporated. The resulting residue was mixed with chloroform and the remaining solid removed by filtration. The solvent was again removed and the last step repeated with cyclohexane. After evaporation of the solvent, 3.18 g (59%) of **6** was obtained as a yellow solid. ^13^C NMR (75.5 MHz, CDCl_3_): 164.1 (1C, C_q_), 155.0 (1C, C_q_), 130.5 (1C, CH), 128.9 (2C, CH), 128.1 (1C, C_q_), 126.5 (2C, CH), 99.5 (1C, CH). ^1^H NMR: 7.77–7.74 (m, 2H, CH), 7.47–7.45 (m, 3H, CH), 6.47 (s, 1H, CH).

**Isoxazole (7a)** [23]**.** Malonaldehyde tetraethyl acetal (22.03 g, 0.1 mol) was added over a 30 min period to hydroxylamine hydrochloride (7.64 g, 0.11 mol) in water (50 mL) at 70 °C. Heating was continued for 3 h and the resulting mixture distilled at 95 °C and a mixture of isoxazole, alcohol and water collected. The distillate was added dropwise to a solution of cadmium chloride (18.30 g) in water (15 mL). The resulting precipitate was filtered, washed with a little cold water and dried. The resulting solid was then suspended in water, heated to boiling and a mixture of isoxazole and water was obtained on distillation. The distillate (two phases) was extracted with ether, dried over magnesium sulfate and filtered. After evaporation of the solvent, 2.97 g (43%) of **7a** was obtained as a colorless liquid. ^13^C NMR (75.5 MHz, CDCl_3_): 157.4 (1C, CH), 148.7 (1C, CH), 103.2 (1C, CH). ^1^H NMR (300 MHz, CDCl_3_): 8.36 (s, 1H, CH), 8.19 (s, 1H, CH), 6.26 (s, 1H, CH).

**5-Methylisoxazole (7b).** A mixture of acetylacetaldehyde (30.0 g, 0.227 mol), diethylamine (17.40 g, 0.238 mol, 24.6 mL) and methanol (70 mL) was heated at 65 °C for 1 h. Hydroxylamine hydrochloride (16.50 g, 0.238 mol) of in water (50 mL) was then added dropwise and heating was continued for 2 h. The solution was cooled to rt, extracted with ether, dried over magnesium sulfate and filtered. The solvent was evaporated and the residue fractionated (78–80 °C, 270 mbar) to yield 4.94 g (26%) of **7b** as a colorless liquid. ^13^C NMR (75.5 MHz, CDCl_3_): 168.4 (1C, C_q_), 150.1 (1C, CH), 100.5 (1C, CH), 11.6 (1C, CH_3_). ^1^H NMR (300 MHz, CDCl_3_): 8.02 (s, 1H, CH), 5.88 (s, 1H, CH), 2.33 (s, 3H, CH_3_).

**3-Methylisoxazole (7c)** [24]**.** A solution of **2** (20.80 g, 0.12 mol) and *p-*toluenesulfonic acid (2.0 g) in chloroform (200 mL) was refluxed for 2 h. The resulting mixture was cooled to rt, washed with aqueous sodium bicarbonate and extracted several times with chloroform. The combined organic phases were washed several times with water, dried over magnesium sulfate, filtered and evaporated in vacuo to give 3.89 g (39%) of **7c** as a colorless liquid. ^13^C NMR (75.5 MHz, CDCl_3_): 158.4 (1C, C_q_), 157.9 (1C, CH), 104.8 (1C, CH), 10.9 (1C, CH_3_). ^1^H NMR (300 MHz, CDCl_3_): 8.22 (s, 1H, CH), 6.11–6.10 (d, 1H, ^3^*J* = 0.9 Hz, CH), 2.24 (s, 3H, CH_3_).

**3,5-Dimethylisoxazole (7d)** [25]**.** A solution of 2,4-pentanedione (100.10 g, 1 mol, 103.2 mL) and hydroxylamine hydrochloride (74.70 g, 1.075 mol) in water (150 mL) and ethanol (100 mL) was refluxed for 90 min. The mixture was cooled to rt, poured onto ice (200 mL) and extracted four times with dichloromethane. The combined organic phases were dried over magnesium sulfate, filtered and evaporated. The resulting dark mixture was fractionated (141 °C) to yield 77.5 g (80%) of **7d** as a colorless liquid. ^13^C NMR (75.5 MHz, CDCl_3_): 168.2 (1C, C_q_), 159.0 (1C, C_q_), 101.6 (1C, CH), 11.1 (1C, CH_3_), 10.4 (1C, CH_3_). ^1^H NMR (300 MHz, CDCl_3_): 5.76 (s, 1H, CH), 2.317 (s, 3H, CH_3_), 2.19 (s, 3H, CH_3_).

**3,4,5-Trimethylisoxazole (7e)** [26]**.** A mixture of **3 (**20.55 g, 0.18 mol), hydroxylamine hydrochloride (12.50 g, 0.18 mol) and water (80 mL) was stirred at rt for 24 h. The solution was then extracted three times with chloroform and the combined organic layers were dried over magnesium sulfate and filtered. After removal of the solvent, the residue was distilled (165 °C) to yield 13.0 g (65%) of **7e** as a colorless liquid. ^13^C NMR (75.5 MHz, CDCl_3_): 163.4 (1C, C_q_), 159.0 (1C, C_q_), 108.2 (1C, C_q_), 9.9 (1C, CH_3_), 9.2 (1C, CH_3_), 5.8 (1C, CH_3_). ^1^H NMR (300 MHz, CDCl_3_): 2.01 (s, 3H, CH_3_), 1.91 (s, 3H, CH_3_), 1.61 (s, 3H, CH_3_).

**3,5-Diphenylisoxazole (7f)** [25]**.** A solution of **4** (4.49 g, 20 mmol) and hydroxylamine hydrochloride (1.74 g, 20 mmol) in water (30 mL) and ethanol (20 mL) was refluxed for 90 min. The mixture was cooled to rt, poured onto ice (50 mL) and extracted two times with dichloromethane. The combined organic phases were dried over magnesium sulfate, filtered and evaporated. The resulting solid was recrystallized from ether to yield 4.09 g (92%) of **7f** as colorless crystals. ^13^C NMR (75.5 MHz, CDCl_3_): 170.3 (1C, C_q_), 162.9 (1C, C_q_), 130.1 (1C, CH), 129.9 (1C, CH), 129.0 (1C, C_q_), 128.9 (2C, CH), 128.8 (2C, CH), 127.4 (1C, C_q_), 126.7 (2C, CH), 125.7 (2C, CH), 97.4 (1C, CH). ^1^H NMR (300 MHz, CDCl_3_): 7.90–7.84 (m, 4H, CH), 7.52 (m, 6H, CH), 6.83 (s, 1H, CH).

**3-Phenyl-5-Methoxy-isoxazole (7g)** [22]**.** A solution of **6** (2.88 g, 16 mmol) and sodium methoxide (7.78 g, 144 mmol) in methanol (40 mL) and THF (40 mL) was heated under reflux for 63 h and then concentrated. The residue was poured in iced water (100 mL), extracted with ether and washed with aqueous sodium carbonate to neutralize hydrochloric acid. The organic layer was dried over magnesium sulfate followed by removal of the solvent. The remaining solid was recrystallized from warm cyclohexane (50 °C) to yield 1.57 g (56%) of **7g** as yellow crystals. ^13^C NMR (75.5 MHz, CDCl_3_): 174.4 (1C, C_q_), 163.9 (1C, C_q_), 129.8 (1C, CH), 129.3 (1C, C_q_), 128.6 (2C, CH), 126.2 (2C, CH), 75.1 (1C, CH), 58.7 (1C, CH_3_). ^1^H NMR: 7.75–7.72 (m, 2H, CH), 7.42–7.39 (m, 3H, CH), 5.51 (s, 1H, CH), 3.96 (s, 3H, CH_3_).

**3-Phenyl-5-(trimethylsilyloxy)isoxazole (7h)** [27]**.** To 2.72 g (25 mmol, 3.2 mL) of chlorotrimethylsilane, was added a mixture of **5 (**3.22 g, 20 mmol) and hexamethyldisilazane (7.26 g, 45 mmol, 9.5 mL). The solution was heated at 120 °C for 30 min and then concentrated. The resulting product was unstable and therefore used immediately.

**NMR-photolyses of isoxazoles 7a**–**h with aldehydes: General procedure.** A solution of isoxazole (0.05 mmol) and aldehyde (0.1 M) in deuterated acetonitrile (0.5 mL) was transferred to a quartz NMR tube, degassed with argon and irradiated in a photo reactor (LZ-C4, 300 nm) for 6 h. The mixture was examined before and after irradiation by ^1^H NMR spectroscopy.

**Photocycloaddition reactions of 7e with aryl substituted aldehydes: General procedure.** A solution of **7e** (2.22 g, 20 mmol) and the corresponding aldehyde (20 mmol) in acetonitrile (200 mL) was transferred to a quartz vessel, mixed with potassium carbonate (0.28 g, 10 mol %) and degassed with nitrogen. The mixture was stirred and irradiated in a Rayonet photochemical reactor (RPR 208, lamps centered 300 nm) at −10 °C for 48–72 h. The resulting yellow solution was concentrated (35 °C) and extracted three times with ether. The combined organic layers were dried over magnesium sulfate and the solvent was evaporated. The solid residue was mixed with a little ethanol, stirred for 30 min, filtered and recrystallized at −10 °C from ethanol.

***exo*-1,4,5-trimethyl-6-phenyl-2,7-dioxa-3-aza-bicyclo[3.2.0]hept-3-en (9a).** Yield: 50%. ^13^C NMR (500 MHz, CD_3_CN): 163.6 (1C, CN), 138.9 (1C, Ar-C), 129.3 (2C, Ar-C), 128.9 (1C, Ar-C), 126.2 (2C, Ar-C), 115.6 (1C, OCO), 86.4 (1C, CO), 63.7 (1C, C_q_), 19.1 (1C, CH_3_), 11.1 (1C, CH_3_), 10.2 (1C, CH_3_). ^1^H NMR (500 MHz, CD_3_CN): 7.45–7.35 (m, 5H, ^3^*J* = 7.6 Hz, ^3^*J* = 7.0 Hz, Ar-CH), 5.59 (s, 1H, CH), 2.05 (s, 3H, CH_3_CN), 1.60 (s, 3H, CH_3_), 0.78 (s, 3H, CH_3_). Anal. calcd. for C_13_H_15_NO_2_: C 71.87, H 6.96, N 6.45. Found: C 71.67, H 6.98, N 6.40. Colorless needles, unit cell parameters: a = 9.3804(6), b = 14.8345(10), c = 9.080(6), β = 116.646(3), space group *P*2_1_/*c*.

***exo*-1,4,5-trimethyl-6-(4'-methylphenyl)-2,7-dioxa-3-aza-bicyclo[3.2.0]hept-3-en (9b).** Yield: 35%. ^13^C NMR (75.5 MHz, CD_3_CN): 163.7 (1C, CN), 138.8 (1C, Ar-C), 136.1 (1C, Ar-C), 130.0 (2C, Ar-C), 126.4 (2C, Ar-C), 115.6 (1C, OCO), 86.6 (1C, CO), 63.8 (1C, C_q_), 21.1 (1C, CH_3_), 19.2 (1C, CH_3_), 11.1 (1C, CH_3_), 10.3 (1C, CH_3_). ^1^H NMR (300 MHz, CD_3_CN): 7.26–7.24 (m, 4H, Ar-CH), 5.55 (s, 1H, CH), 2.35 (s, 3H, Ar-CH_3_), 2.03 (s, 3H, CH_3_CN), 1.60 (s, 3H, CH_3_), 0.79 (s, 3H, CH_3_). Anal. calcd. for C_14_H_17_NO_2_: C 72.70, H 7.41, N 6.06. Found: C 72.71, H 7.45, N 6.08. Colorless needles, unit cell parameters: a = 17.196(2), b = 5.9199(4), c = 12.807(2), β = 109.944(4), space group *P*2_1_/*c*.

***exo*-1,4,5-trimethyl-6-(3'-methylphenyl)-2,7-dioxa-3-aza-bicyclo[3.2.0]hept-3-en (9c).** Yield: 65%. ^13^C NMR (75.5 MHz, CD_3_CN): 163.7 (1C, CN), 139.3 (1C, Ar-C), 139.0 (1C, Ar-C), 129.7 (1C, Ar-C), 129.3 (1C, Ar-C), 126.9 (1C, Ar-C), 123.4 (1C, Ar-C), 115.7 (1C, OCO), 86.6 (1C, CO), 63.8 (1C, C_q_), 21.4 (1C, CH_3_), 19.2 (1C, CH_3_), 11.1 (1C, CH_3_), 10.3 (1C, CH_3_). ^1^H NMR (300 MHz, CD_3_CN): 7.35–7.13 (m, 4H, Ar-CH), 5.54 (s, 1H, CH), 2.37 (s, 3H, Ar-CH_3_), 2.04 (s, 3H, CH_3_CN), 1.60 (s, 3H, CH_3_), 0.79 (s, 3H, CH_3_). Anal. calcd. for C_14_H_17_NO_2_: C 72.70, H 7.41, N 6.06. Found: C 72.55, H 7.39, N 6.09. Colorless needles, unit cell parameters: a = 13.3045(8), b = 6.9356(6), c = 17.5348(10), β = 129.284(3), space group *P*2_1_/*c*.
